# Aberrated surface soliton formation in a nonlinear 1D and 2D photonic crystal

**DOI:** 10.1371/journal.pone.0194632

**Published:** 2018-03-20

**Authors:** Vyacheslav A. Trofimov, Tatiana M. Lysak, Evgenii M. Trykin

**Affiliations:** Faculty of Computational Mathematics and Cybernetics, Lomonosov Moscow State University, Moscow, Russia; Oregon State University, UNITED STATES

## Abstract

We discuss a novel type of surface soliton—aberrated surface soliton—appearance in a nonlinear one dimensional photonic crystal and a possibility of this surface soliton formation in two dimensional photonic crystal. An aberrated surface soliton possesses a nonlinear distribution of the wavefront. We show that, in one dimensional photonic crystal, the surface soliton is formed at the photonic crystal boundary with the ambient medium. Essentially, that it occupies several layers at the photonic crystal boundary and penetrates into the ambient medium at a distance also equal to several layers, so that one can infer about light energy localization at the lateral surface of the photonic crystal. In the one dimensional case, the surface soliton is formed from an earlier formed soliton that falls along the photonic crystal layers at an angle which differs slightly from the normal to the photonic crystal face. In the two dimensional case, the soliton can appear if an incident Gaussian beam falls on the photonic crystal face. The influence of laser radiation parameters, optical properties of photonic crystal layers and ambient medium on the one dimensional surface soliton formation is investigated. We also discuss the influence of two dimensional photonic crystal configuration on light energy localization near the photonic crystal surface. It is important that aberrated surface solitons can be created at relatively low laser pulse intensity and for close values of alternating layers dielectric permittivity which allows their experimental observation.

## 1. Introduction

Among the various problems of laser pulse interaction with a photonic crystal (PC), the soliton formation and light localization are very interesting problems [1–19] partially due to the possibility of their wide use in information technologies. For example, recent investigations [20–23] are devoted to soliton formation in a one-dimensional photonic lattice across a graphene monolayer. In particular, investigation of the soliton interaction with dissipative waves plays an important role in our understanding of supercontinuum generation [24–26]. Surface modes at the edge of a semi-infinite chirped photonic lattice have been studied theoretically [27].

In a photonic crystal, which is characterized by the periodic variation of their properties in one direction only, soliton propagation along the nano-layers is of special interest. Such propagation was analyzed recently [28] on the basis of Maxwell's equations, where the existence and stability of TM and TE spatial solitons were investigated. Light propagation in such structures was also analyzed both experimentally and numerically in [29–32]. Nonlinear interaction of femtosecond pulses with one dimensional (1-D) photonic crystal (layered structure) was investigated [33–35], where the soliton formation in certain layers with cubic nonlinear response was considered. This soliton appears due to cubic nonlinear response in a layer of photonic crystal under certain condition between the wavelength of the optical radiation and the thickness of the photonic crystal. Showing the twenty times intensity growth in photonic crystal [34], we explained the photonic crystal damage, observed in experiments. In [36], the similar color soliton was generated if two waves with basic and doubled frequencies interact.

Two dimensional (2-D) solitons of various types can exist in the photonic crystal with properties varying in two axes. Among them, there are fundamental solitons, vortex solitons and gap solitons [37–41]. Their stability in photonic lattices is discussed, for example, in [38, 42]. The role of photonic crystal defect in vortex solitons generation is revealed [43], and the possibility of photonic crystal cladding to stabilize spatial two dimensional solitons is discussed in [44].

A very important part of modern investigations is the formation of surface solitons at a laser pulse propagation in a photonic crystal and this investigation has been pursued for many years. For example, formation of one dimensional gap solitons is reported in [45–53]. The existence of the vortex solitons at the edge and in the corners of two dimensional triangular photonic lattice is shown in [54]. Two dimensional solitons at the interface between square and hexagonal waveguide arrays were experimentally observed in [55]. The possibility of formation of two color surface solitons in the corners or at the edges of a square photonic lattice is discussed in [56]. In all these papers, the soliton occupies a single boundary layer or several solitons occupy several layers which are close to the photonic crystal faces. It should be stressed that if several solitons occupy several layers, a number of zero intensity points can be clearly seen in their intensity distribution. The penetration of such solitons into the ambient medium is small, and the solitons do not change their location during their propagation along the photonic lattice or photonic structure.

In this paper, on the base of computer simulation, we demonstrate a possibility of a novel type surface soliton—aberrated surface soliton—appearance in one dimensional photonic crystal. The surface soliton is formed at the photonic crystal boundary with the ambient medium. The novelty of this soliton is its transversal size and position, and nonlinear distribution of the wave front. It is very important that only a part of the one dimensional surface soliton localizes in the photonic crystal and can occupy its several layers. The other its essential part localizes near the boundary outside the photonic crystal. Hence, one can tell about the light energy localization at the lateral surface of the photonic crystal. The solitons appear in the layered structure with alternating layers with high and low dielectric permittivity. The layers with large refractive index are linear or possess small focusing nonlinearity in comparison with the focusing nonlinearity of the layers with small refractive index. It is important that the aberrated surface solitons can be created at relatively low laser pulse intensities and for close values of alternating layers dielectric permittivity which allows their experimental observation.

It should be stressed once again that the main difference of the surface soliton under consideration from the solitons, investigated in [45–53], for example, is that the surface soliton under consideration occupies several layers at the photonic crystal boundary and penetrates into the ambient medium at the distance of several layers. Each of the solitons described in those papers occupies only negligible part of the ambient medium. Moreover, those solitons consist of numerous sub-solitons. Our soliton possesses a smooth Gaussian-like profile. In this paper, we also describe the process of the considered surface solitons formation from the earlier formed soliton that falls on the photonic crystal at the angle different from the normal to the photonic crystal face (which means the presence of the transverse component of the soliton wave vector).

It should be noted that in this paper we continue and extend our results on one dimensional surface solitons which we have reported earlier [57, 58]. In particular, we show the possibility for such soliton appearance for a wide range of PC parameters and laser radiation parameters and discuss the oscillating narrow surface soliton formation. We show that the surface soliton under consideration possesses a nonlinear wavefront distribution: so it is an aberrated soliton. We also present here our new results on the energy localization in a two dimensional photonic crystal. In our opinion, the mechanism of energy localization in a two dimensional photonic crystal is similar to the surface soliton formation in a one dimensional photonic crystal. That is why we report our results on one- and two dimensional photonic crystals together in the present paper.

The paper is organized as follows. In Section 1 we present the problem statement for laser pulse propagation in the photonic crystal under consideration. Section 2 deals with the investigation of the one dimensional surface soliton formation for various laser pulse incident intensities and photonic crystal optical properties. In particular, we consider the photonic crystal with linear layers alternating with the focusing layers due to cubic nonlinear response and the photonic crystal with alternating focusing layers with different cubic susceptibility. We discuss in detail the process of the one dimensional surface soliton formation and show the ambient medium properties influence on its appearance. Section 3 is devoted to the possibility of the two dimensional soliton formation in the photonic crystal consisting of spheres if the incident Gaussian laser pulse falls at the photonic crystal face. We consider three different relations between the photonic crystal face size and the incident beam radius, and show that the light localization takes place in the photonic crystal in all these cases.

## 2. Statement of the problem for laser pulse propagation in PC

**Fig 1:**
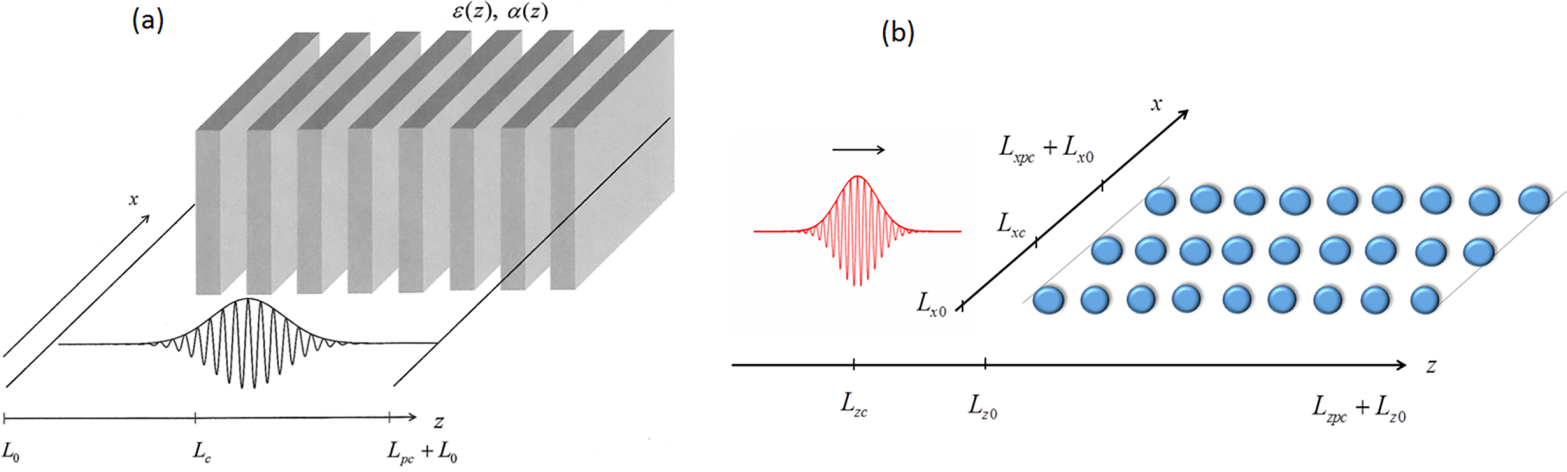
Scheme for laser pulse interaction with 2-D PC, consisting of layers (a) or spheres (b).

We start our consideration from the soliton propagating in 2-D PC along the layers of PC (Fig 1A) or along the rows of spheres (Fig 1B). As it is well-known, the femtosecond pulse propagation in 2-D PC is governed by the following wave equation:

$$\begin{array}{l} \frac{\partial^{2}E(z,x,t)}{\partial z^{2}}+\frac{\partial^{2}E(z,x,t)}{\partial x^{2}}-\frac{n^{2}(z,x)}{c^{2}}\frac{\partial^{2}E(z,x,t)}{\partial t^{2}}=\frac{4\pi }{c^{2}}\frac{\partial^{2}}{\partial t^{2}}P_{nl},\\ P_{nl}=\chi^{\left(3\right)}|E{|}^{2}E\;,\;0<t<L_{t}\;,\;0<z<L_{z},\;0<x<L_{x} \end{array}$$ (1)

where *E*(*z*,*x*,*t*) is the electric field strength; x is a coordinate, along which the laser pulse propagates and *L*_*x*_ - its maximum value; z is a transverse coordinate and *L*_*z*_ is its maximum value; $n(z,x)=\sqrt{\varepsilon (z,x)}$ is a medium refractive index; *t* is time and *L*_*t*_ is a time interval, within which the pulse propagation is analyzed; c is a light velocity in vacuum; *χ*^(3)^ is a cubic susceptibility of PC’s layers. Thus we consider propagation of the pulse with incident finite distribution during limited time interval.

Under the description of laser pulse propagation in the PC we take into account the beam diffraction along all space coordinates which allow describing the propagation of the waves reflected from each of the PC layers faces. However, we use a slowly varying envelope approximation in the time coordinate. This approach was proposed in [59, 60]. We neglect the second order dispersion but take into account the dependence of the group velocity on the layers refractive indexes. Consequently, the electric field strength and nonlinear medium response can be written in the form

$$E(z,x,t)=0.5E_{0}(A(z,x,t)e^{-i\omega t}+c.c.)\;,\;P_{nl}=\chi^{\left(3\right)}{\left|A\right|}^{2}0.125E_{0}^{3}(A(z,x,t)e^{-i\omega t}+c.с.).$$

Here *A*(*z,x,t*) is a slowly varying amplitude in time only; *c.c.* denotes a conjugation of complex function, *E*_0_ is amplitude of the electric field strength. Assuming a linear relationship between wave number *k* and frequency of light *ω*, as it is usual for this kind of problem, and using the same coordinate’s notations for convenience, one can get from the wave Eq (1) the following nonlinear Schrödinger equation, which is written in dimensionless variables

$$\varepsilon (z,x)\frac{\partial A}{\partial t}+iD_{z}\frac{\partial^{2}A}{\partial z^{2}}+iD_{x}\frac{\partial^{2}A}{\partial x^{2}}+i\beta \left[\varepsilon (z,x)+\alpha (z,x){\left|A\right|}^{2}\right]\;A=0,\;t>0\;,\;0<z<L_{z},\;0<x<L_{x},$$ (2)

where

$$\begin{array}{l} \varepsilon (z,x)=n^{2}(z,x),\;\alpha (z,x)=3\pi \chi^{\left(3\right)}(z,x)E_{0}^{2},\\ D_{z}=-\frac{1}{4\pi \Omega },\;\beta =-\pi \Omega ,\;\Omega =\frac{\omega }{\omega_{str}}=\frac{\lambda_{0}}{\lambda },\;\omega_{str}=2\pi c/\lambda_{0}. \end{array}$$ (3)

Above, *E*_0_ is the incident pulse amplitude, *ω*_*str*_ is a frequency and *λ*_0_ a characteristic length of the periodic structure. The last two parameters, as well as dimensionless parameter *D*_*x*_ and dimensionless variables *z* and *t* depend on the PC characteristic structure and will be specified in Sec.2 and 3 for the schemes of laser interaction with PC (Fig 1).

Note that the validity of Eq (2) is limited by the pulse duration: this duration should not be less than some values that are determined by the second order dispersion. It is important to stress that taking into account the second order derivative along the space coordinates, Eq (2) is valid for description of fast oscillating processes along this coordinates as well as for the beam reflection from the PC layers faces because we do not use the slowly varying envelope approximation for the space coordinates.

## 3. Localization of the oscillating soliton near the boundary of a 1-D nonlinear layered photonic crystal

Characteristic length of the periodic structure, parameter *D*_*x*_ and dimensionless variables *z* and *t* of Eq (2) for the laser pulse interaction with 2-D PC, consisting of layers (Fig 1A), are the following:

$$\begin{array}{l} D_{x}=-D_{z}\frac{\lambda_{0}^{2}}{a_{x}^{2}},\;\lambda_{0}=d_{1}\sqrt{\varepsilon_{1}}+d_{2}\sqrt{\varepsilon_{2}},\\ z\to \frac{z}{\lambda_{0}},\;x\to \frac{x}{a_{x}},\;t\to t\frac{c}{\lambda_{0}},\;L_{z}\to \frac{L_{z}}{\lambda_{0}},\;L_{x}\to \frac{L_{x}}{a_{x}},\;L_{t}\to \frac{cL_{t}}{\lambda_{0}}. \end{array}$$

Above parameter *a*_*x*_ characterizes the laser beam profile on the x coordinate. The other dimensionless parameters are specified in Eq (3).

For simplicity at the first stage of investigation we consider a 1-D PC taking into account its homogeneity along the x coordinate, i.e. we neglect the dependence of complex amplitude and functions *ε*(*z*,*x*), *α*(*z*,*x*) on the x coordinate:

$$\begin{array}{l} A(z,x,t)\to A(z,t),\\ (\varepsilon (z),\alpha (z))=\left\{ \begin{array}{l} (\varepsilon_{3},\alpha_{0}),\;0\leq z\leq L_{0},\\ (\varepsilon_{1},\alpha_{1}),\;0\leq z-L_{0}-(d_{1}+d_{2})(j-1)\leq d_{1},\;1<j<N_{str}+1,\\ (\varepsilon_{2},\alpha_{2}),\;0\leq z-L_{0}-d_{1}-(d_{1}+d_{2})(j-1)\leq d_{2},\;1<j<N_{str},\\ (\varepsilon_{3},\alpha_{0}),\;L_{0}+L_{pc}\leq z\leq L, \end{array}\right. \end{array}$$ (4)

*ε*_3_ and *α*_0_ is the dielectric permittivity and cubic nonlinearity of ambient medium, respectively. The negative value of *α*_0_, *α*_1_, *α*_2_ results in the self-focusing of laser beam. *d*_1_, *d*_2_, *ε*_1_, *ε*_2_ and *α*_1_, *α*_2_ are thicknesses, dielectric permittivity and nonlinearity coefficients of alternating layers correspondingly; *N*_*str*_ is a number of layers pairs. *L* is the normalized length of the considered region along the z coordinate (it includes the distance *L*_0_ before the PC, *L*_*pc*_ - length of layered structure, and *L*_0_ - length after the PC as well, see Fig 1.

Eq (2) under the condition of *D*_*x*_ = 0 is solved with the following artificial boundary conditions:

$$\begin{array}{l} \frac{\partial A}{\partial t}-\frac{1}{\sqrt{\varepsilon (z)}}\frac{\partial A}{\partial z}+i2\beta A=0\;,\;t>0\;,\;z=0,\\ \frac{\partial A}{\partial t}+\frac{1}{\sqrt{\varepsilon (z)}}\frac{\partial A}{\partial z}+i2\beta A=0\;,\;t>0\;,\;z=L. \end{array}$$ (5)

The incident complex amplitude may be written in the form

$$A(z,t)|{}_{t=0}=A_{s}(z)\exp(-i2\pi \Omega_{z}(z-L_{c}))$$ (6)

for investigation of the influence of transverse perturbations. Here *A*_*s*_(*z*) is the soliton profile, which is in the PC. The algorithm for the soliton finding is described in [61,62]. The exponential factor in (6) describes the perturbation of laser beam propagation direction. Parameter *L*_*c*_ is the coordinate of the soliton center along the z-coordinate (see Fig 1).

In our computer simulation we consider the soliton propagating along the layers of PC (Fig 1A) at a perturbation of the propagation direction due to the presence of transverse component of wave vector (6). In other words, the propagation of soliton, which falls on the layered structure at the angle differing from normal to the PC surface, is investigated. The layered structure consists of many layers and the soliton under consideration is also spreading along the number of them.

All the incident solitons that we consider below in this paper are stable with respect to small transverse perturbations of the wave vector (Eq (6), Ω_*z*_ < 0.01). This means that such small perturbation do not cause the soliton motion along the transversal coordinate, and the soliton profile remains the same. Larger transverse perturbations can result in the soliton motion along the transversal coordinate. Nevertheless, the soliton profile remains approximately the same, slightly changing as the soliton intensity maximum passes through alternating layers.

In section 3.1 we describe the details of the aberrated surface soliton formation. Sections 3.2 and 3.3 report about the influence of the laser radiation, and the PC layers, and ambient medium optical properties on the localized soliton features.

### 3.1 Details of an aberrated surface soliton formation

The details of the oscillating soliton formation were described in [57] for a PC with 42 layers with the same dimensionless length *d*_1_ = *d*_2_ = 0.289 and the following set of the PC dimensionless parameters: Ω = 10.382, *α*_1_ = 0, *α*_2_ = −10^−3^ (this sign of nonlinear coefficient corresponds to laser beam self-focusing), *ε*_1_ = 5, *ε*_2_ = 1.5, *ε*_3_ = 1, and linear ambient medium (*α*_0_ = 0). These dimensionless parameters correspond to light wavelength *λ* = 1 *μm*; thickness of layers *d*_1_ = *d*_2_ = 3 *μm*, and characteristic wavelength of layered structure *λ*_0_ ≈ 10.382 *μm*. The power density of optical radiation is equal to 10 MW/cm^2^ or several tens MW/cm2—which is normal for femtosecond laser pulse. It should be noted that the dielectric permittivity values are measured in the units of the ambient medium permittivity, and they are close to the ones for a semiconductor PC. Thus, odd layers are linear ones, while even layers contain the cubic nonlinearity. In the case under consideration, the first layer, which corresponds to the smallest value of the z-coordinate, is a linear one and the last layer is a nonlinear one.

**Fig 2:**
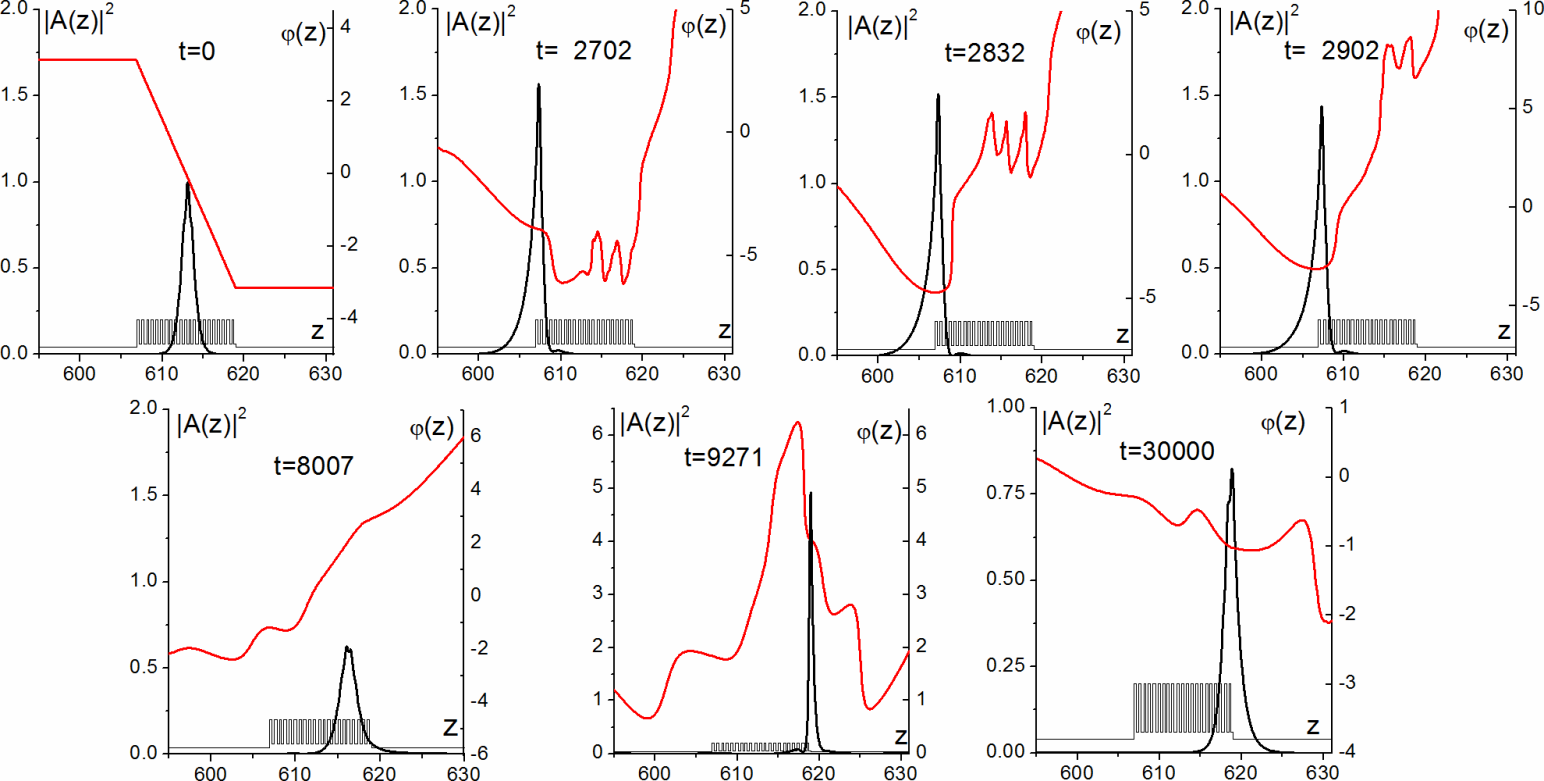
Soliton profile evolution (black curves) and beam phase evolution (red curves) at various time moments (shown in figures) for Ω = 10.382, *α*_0_ = 0, *α*_1_ = 0, *α*_2_ = −10^−3^, *ε*_1_= 5, *ε*_2_ = 1.5, *ε*_3_ = 1, *d*_1_ = *d*_2_ =0.289 and Ω_*z*_ = 0.082. PC layers are also depicted in the figure.

**Fig 3:**
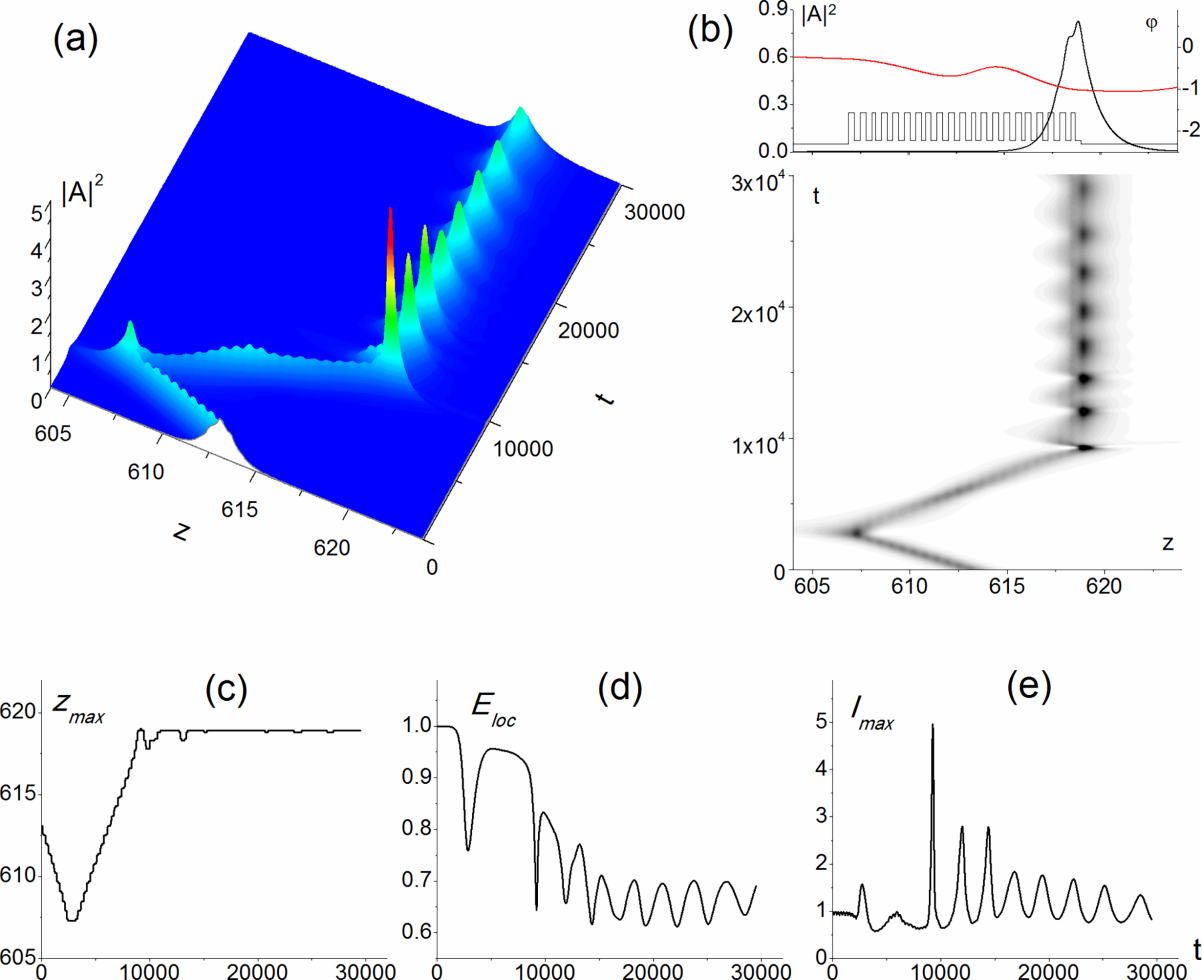
Formation of the surface soliton (a,b). Position of maximal intensity (c) and localized energy (d) versus time. Value of maximal intensity versus time (e). Parameter values are Ω = 10.382, *α*_0_ = 0, *α*_1_ = 0, *α*_2_ = −10^−3^, *ε*_1_= 5, *ε*_2_ = 1.5, *ε*_3_ = 1, *d*_1_ = *d*_2_ =0.289 and Ω_*z*_ = 0.082. A snapshot at the top figure (b) corresponds to the soliton profile (black line) and phase distribution (red line) at the time moment *t* = 30000.

The soliton profile *A*_*s*_(*z*) is shown in Fig 2 in the time moment t = 0. It is formed inside the PC and is spreading over about 17 layers. As it was already mentioned, the soliton is stable with respect to small transverse perturbations of the wave vector (formula (6), Ω_*z*_ < 0.01). Nevertheless, the wave vector large perturbation (Ω_*z*_ in the range from 0.04 to 0.09) along the transverse coordinate results in the soliton motion and, as a result, the oscillating soliton is formed near the right boundary of the PC. For example, it occurs if the wave vector projection is equal to Ω_*z*_ = 0.082 (Figs 2 and 3). In this case, the soliton evolution occurs into two stages. At the first stage the soliton moves towards the left boundary of PC. Then it reflects from the boundary and propagates to the right boundary (Fig 2, t = 2832, 8007, Fig 3). It should be emphasized that about 20% of soliton energy temporally leaves the PC at the reflection (Fig 3D), while the maximum intensity grows 1.5 times (Fig 2, t = 2832, Fig 3C and Fig 3E) in comparison with its initial value. At the second stage, the narrow soliton stops at the right boundary and it oscillates (Fig 2, t = 9271). This soliton is located primarily in the last PC layer with dielectric permittivity *ε*_2_ = 1.5 and nonzero cubic nonlinearity *α*_2_ = −10^−3^. Its spatial size is at least 4 times less than the initial one, and the maximum intensity increases up to 5 times.

Further evolution of the soliton is characterized by the maximum intensity oscillations damping so that the maximum intensity decreases gradually up to 1 dimensionless unit at the end of time interval (Fig 2, t = 30000, Fig 3E). During the whole time under consideration the maximum intensity belongs to the last nonlinear layer. It is very interesting, that only about 60% of total energy of the oscillating soliton remains inside the PC. The other part of its energy is localized in the substrate near the boundary of PC (Fig 2, t = 30000, Fig 3D).

In order to explain the soliton formation we also show phase distribution (red curves in Fig 2). As it is well seen, the soliton motion direction is defined by the phase gradient sign. If the phase gradient is negative (phase decreases with coordinate increasing) at the initial time moment, then the soliton starts moving to the left (Fig 2, t = 0). The opposite situation is shown for t = 8007 in Fig 2, when the soliton moves to the right. As the soliton reaches the left boundary, it slows down, and the absolute value decreasing for the phase gradient in the area of the pulse maximal intensity takes place (Fig 2, t = 2702). Then, the phase minimum is formed at the location of the pulse maximal intensity (Fig 2, t = 2832). Furthermore, the pulse evolution is characterized by the phase decreasing to the left of the gradient minimum (Fig 2, t = 2902), so that, shortly after that time, the phase gradient becomes positive in the soliton area. As a result, the soliton motion direction changing takes place. At the right PC boundary (Fig 2, 30000), one part of the soliton tends to go left and the other part tends to go right, because the phase minimum is formed at the location of the pulse maximal intensity. So, the soliton stops and one can say that the aberrated surface soliton is formed due to the nonlinear distribution of the soliton wavefront. Small oscillations of the maximal intensity position at the right boundary of the PC (Fig 3C) can also be explained by phase distribution analysis (Fig 2, t = 9271).

### 3.2 Influence of laser radiation parameters and optical properties of PC layers on the aberrated surface soliton formation

It is very important to stress that surface soliton formation takes place for a wide range of PC and laser radiation parameters. In particular, we observed this phenomenon for the focusing cubic nonlinearity of the even layers from the range 10^−4^ ≤ |*α*_2_| ≤ 2.5, dielectric permittivity of alternating layers varying from 5 to 1 dimensionless units, thickness of the layers from 0.1 to 3 *μm* and the light wavelength from 0.5 to 1.25 *μm*. Corresponding power density of optical radiation varies from several tens MW/cm^2^ to several tens GW/cm^2^. Essentially, we also observed this phenomenon for the alternating layers with close focusing nonlinearity and dielectric permittivity (see Section 3.3).

The main conclusion of our consideration is that the surface soliton can be formed for the limited range of the wave vector transverse perturbations. This range strongly depends on the strength of the focusing cubic nonlinearity (the power density of laser radiation), the ratio $\Omega =\frac{\lambda_{0}}{\lambda }$ and on the nonlinearity of the ambient medium. (The influence of the ambient medium will be discussed below in Section 3.3.) So, the smaller is the power density of laser radiation, the less values of parameters Ω and Ω_*z*_ are required for the surface soliton formation.

**Fig 4:**
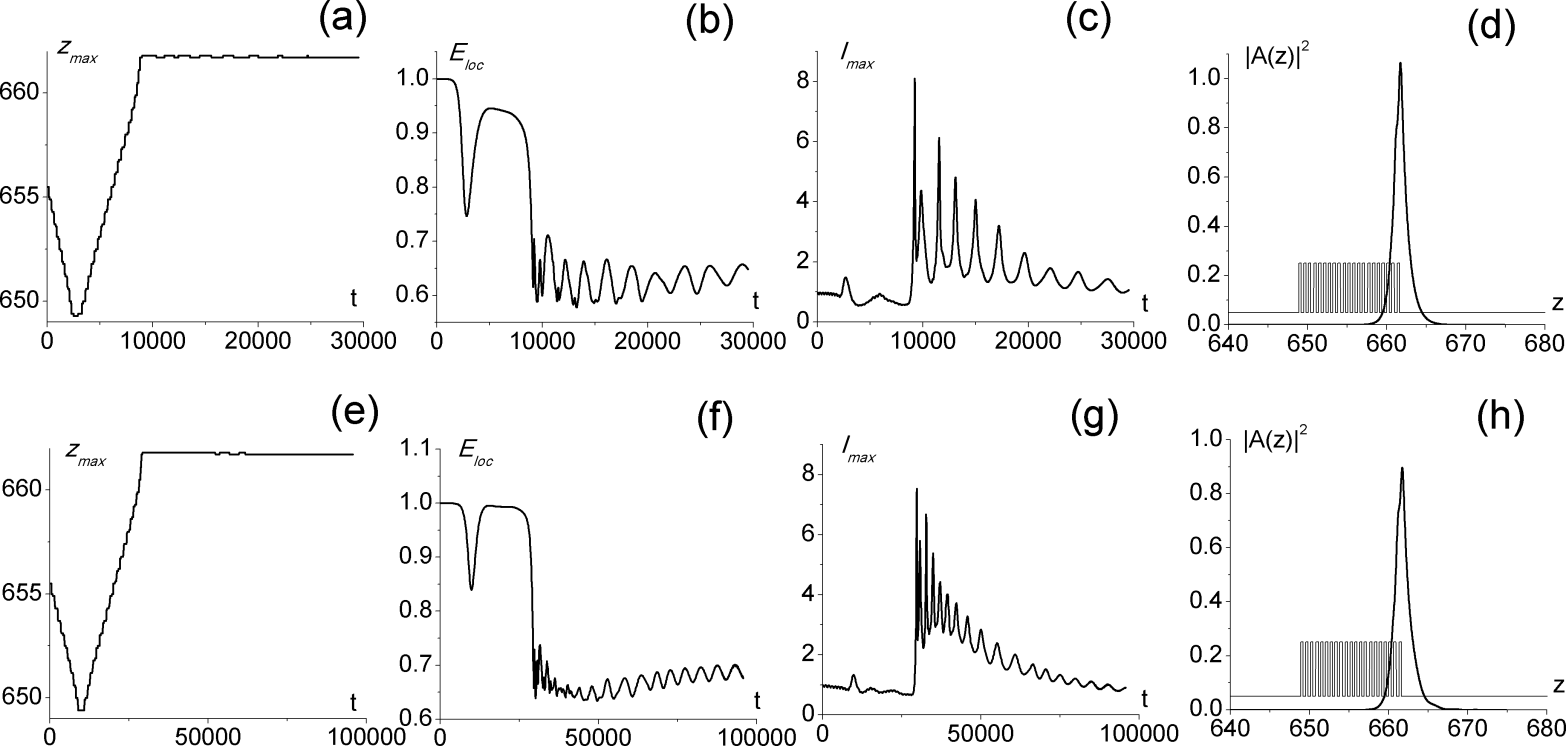
Maximal intensity position (a,e), localized energy (b,f) and maximal intensity (c,g) versus time. Soliton shape at the end of time interval t = 30000 (d,h) calculated for parameters Ω = 9.708, *α*_2_ = −10^−3^, and Ω_*z*_ = 0.077 (a-b) or Ω = 19.416, *α*_2_ = −0.25 ⋅ 10^−3^ and Ω_*z*_ = 0.0385 (e-h). Other parameter values are *α*_0_ = 0, *α*_1_ = 0, *ε*_1_ = 5, *ε*_2_ = 1, *ε*_3_ = 1, *d*_1_ = *d*_2_ = 0.309. Linear layers are also depicted in the figure.

As it was already mentioned, for the parameter values discussed in Section 3.1 (*α*_2_ = −10^−3^), the wave vector transverse perturbations should belong to the range 0.04–0.09. Larger perturbations (0.09–0.12) results in the moving soliton formation inside the PC or in the exit of the most part of the soliton energy outside the PC (if transverse perturbations are bigger than 0.12). The change of the dielectric permittivity of the even layers from *ε*_2_ = 1.5 to *ε*_2_ = 1 slightly influences the surface soliton formation (in Fig 4A–4D, we used a slightly smaller perturbation Ω_*z*_ = 0.077). The decrease of the absolute value of the even layers cubic focusing nonlinearity up to |*α*_2_| = 0.25 ⋅ 10^−3^ (it means the maximal intensity decreasing for the incident laser beam) requires the light wavelength decreases two fold (up to *λ* = 0.5 *μm*) for the formation of the surface soliton with the same intensity and width. In this case, a two times decrease of the transverse perturbation (up to Ω_*z*_ = 0.0385) is also necessary (Fig 4E–4H). However, the time necessary for the surface soliton formation increases up to 3 times and a slight increasing of localized energy (from ≈0.64 to ≈0.68) also takes place.

Moreover, for all the cases discussed above, the profile of the soliton, localized at the PC boundary, is similar to the initial profile. Their maximal intensity and width are close each other. The maximal intensity position for the surface soliton is between the last right layer and its neighboring layer. So, the surface soliton penetrates into the PC at the distance of ≈ 1.2 dimensionless units (about 4 layers), which corresponds to 12.5 *μm*, and it goes beyond the PC into the substrate at the distance of 0.6 dimensionless units (about 2 layers), which corresponds to 6.3 *μm*. These estimations are made for the full-width at half-maximum (FWHM) of the beam. The whole area, occupied by the soliton outside the PC, can be estimated as 9 layers (≈ 2.8 dimensionless units), while the whole area occupied by the soliton inside the PC, can be estimated also as 9 layers for the first set of parameters under consideration, for example.

Beside the broad multi-layer surface solitons, a narrow oscillating soliton can be formed at the PC face due to transverse perturbations of the wave vector. Indeed, increase of the laser radiation power density from several tens MW/cm^2^ to several tens GW/cm^2^ (increase of the absolute value of *α*_2_ from 10^−3^ to 2.5) and simultaneous decrease of the PC layers thickness (from 3 *μm* to 0.1 *μm*) results in the narrow oscillating soliton formation at the right PC boundary if the light wavelength is *λ* = 1.25 *μm* and the transverse wave number perturbation is Ω_*z*_ = 0.077. In this case, the aberrated surface soliton with a period of oscillation being equal to several dimensionless units is formed at the time moment about 216 dimensionless units which is about 1000 times smaller than for the laser radiation with less power density (less value of the coefficient *α*_2_). This is because the ratio of characteristic wavelengths of the corresponding layered structures is equal to 0.032. Nevertheless, the soliton formation process is similar to the previous case. This soliton localizes mainly in the last PC layer, which is characterized by dielectric permittivity equal to 1 (*ε*_2_ = 1) and non-zero cubic nonlinearity *α*_2_ = −2.5. The neighboring left layer is a linear one (*α*_2_ = 0) with *ε*_1_ = 5.

**Fig 5:**
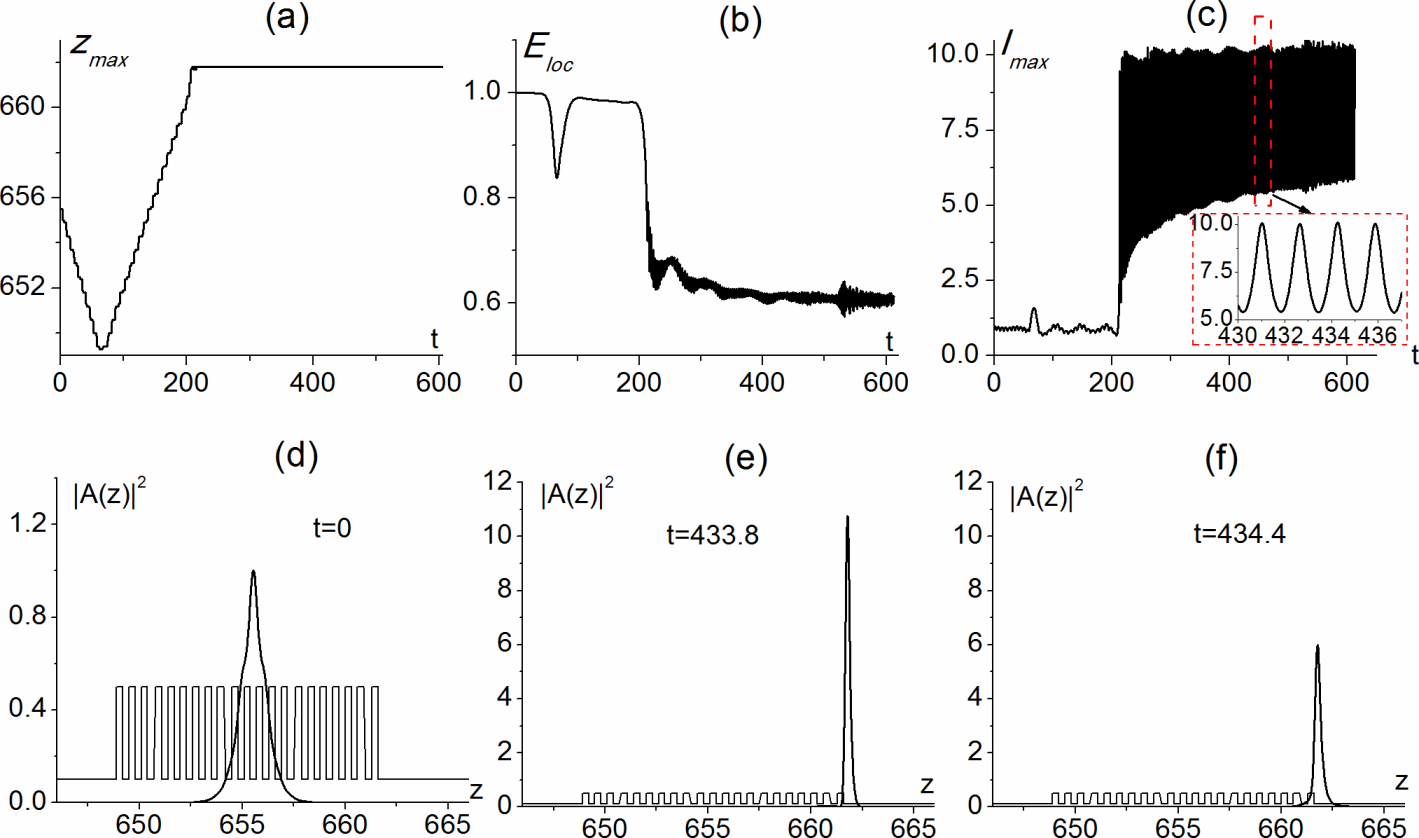
Maximal intensity position (a), localized energy (b) and maximal intensity (c) versus time) and soliton shape at different times (d-f, time moments are shown in figures) calculated for parameters Ω = 0.258, *α*_0_ = 0, *α*_1_ = 0, *α*_2_ = −2.5, *ε*_1_ = 5, *ε*_2_ = 1, *ε*_3_ = 1, *d*_1_ = *d*_2_ = 0.309 and Ω_*z*_ = 0.077.

The oscillating soliton is at least 4 times narrower than the initial intensity profile and its maximal intensity is 5 to 10 times greater in comparison with the initial one. The period of oscillation is about 1.6 dimensionless units (insert in Fig 5C). The FWHM of the oscillating soliton is about the length of one layer (Fig 5E and 5F). So, in this case, the distance of soliton penetrating inside the PC, as well as the distance of its penetrating into substrate, does not exceed one layer. Similar to the previous cases, about 60% of total energy of the oscillating soliton remains inside PC. The other 40% of energy is localized in the substrate in the nearest vicinity of PC (Fig 5B).

It should be noted that the main difference of the surface soliton, described above, from the surface solitons, investigated in [46, 48–51], is the absence of sub-solitons (absence of zero intensity points or intensity minima points in the intensity profile) and aberrated distribution of its phase. One part of the aberrated soliton is localized in the last PC layer with focusing nonlinearity, while the other is in the substrate. Strong intensity oscillations take place for this type of aberrated surface soliton. It should be noted that in discrete systems [46,48], multiple sub-solitons can be partly or completely distinguished. These sub-solitons are localized in the PC altering layers. Surface gap solitons, which exist at the interface between uniform media and periodic medium with Kerr-type cubic defocusing nonlinearity, also consist of a number of separate sub-solitons [49–51]. We believe that the physical mechanism of multi-soliton appearance is the presence of the coupling between the separate elements of discrete arrays. Due to this coupling, a part of the soliton energy from the given element of the array is transferred to the neighboring elements. The presence of intensity minima between these solitons depends on the distance between the neighboring elements and the strength of the coupling. In certain case, one can observe the separate solitons. It is our opinion, that in our case, such a soliton structure can be the result of convective modulation instability of the incident beam propagating in the PC with cubic nonlinear response.

### 3.3 Control of the surface oscillating soliton formation in PC with all nonlinear layers due to the ambient medium properties variation

This section is devoted to the possibility of the control of the aberrated surface soliton formation due to the variation of the ambient medium properties. We also show that this soliton can be formed in the PC with all nonlinear focusing layers. With this aim, we represent some results from our previous paper [58].

**Fig 6:**
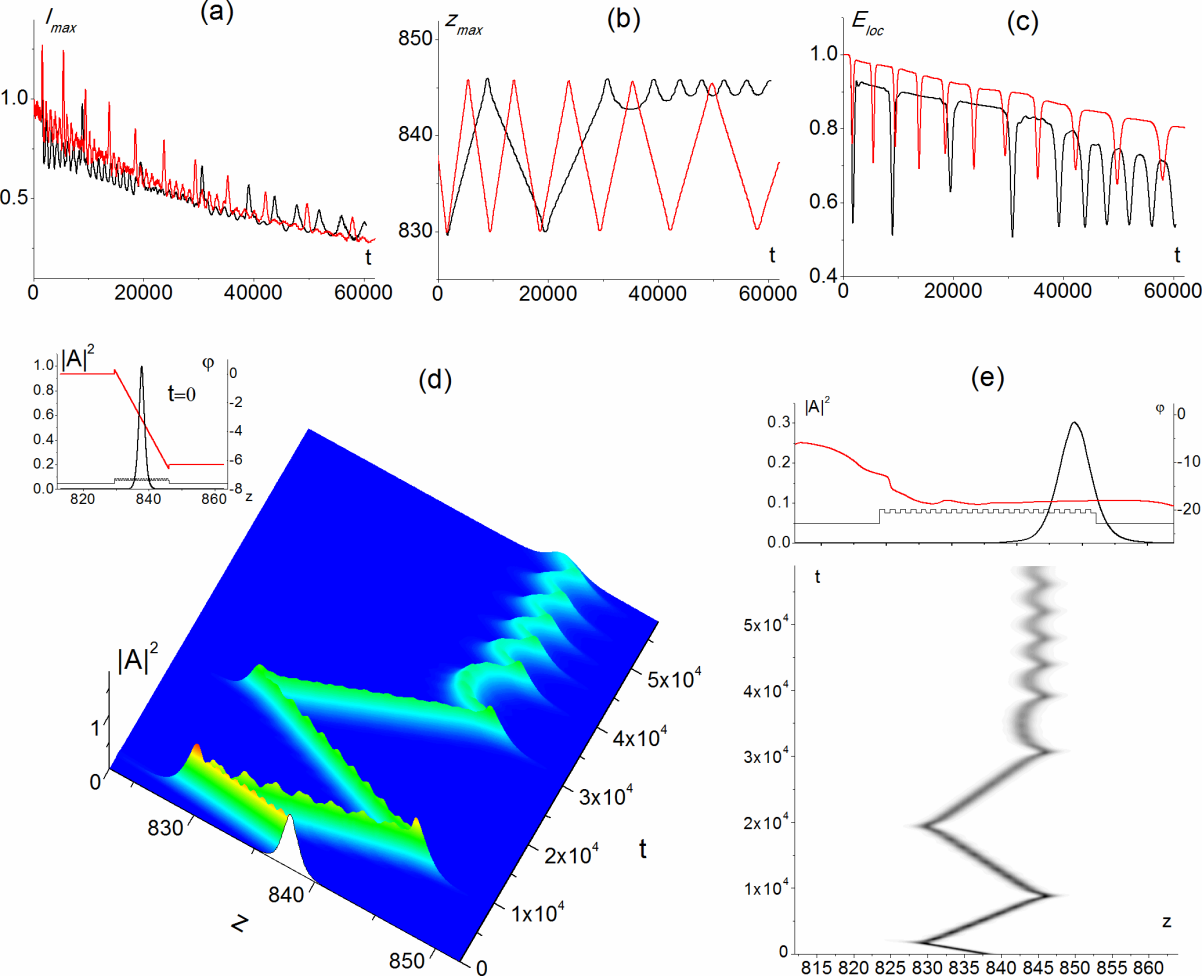
Maximal intensity (a, black line), its position (b, black line) and energy localized in PC (c, black line) versus time, surface soliton formation (d, e) in PC with 42 layers for parameters Ω = 7.5858, *α*_1_ = −10^−3^, *α*_2_ = −1.5 ⋅ −10^−3^, *ε*_1_ = 1.7, *ε*_2_ = 1.5, *ε*_3_ = 1, Ω_*z*_ = 0.0663 and focusing (*α*_0_ = −0.5 ⋅ 10^−3^) nonlinear ambient medium. Red lines in figures (a)-(c) corresponds to the linear (*α*_0_ = 0) ambient medium. Snapshot at the top of figure (e) corresponds to the soliton profile (black line) and soliton phase distribution (red line) at the end of the propagation distance. Insert in figure (d) shows the initial soliton profile (black line) and phase distribution (red line) at t = 0. PC layers are also depicted in the figure.

We consider PC and laser radiation with characteristics close to the ones discussed in sections 3.1 and 3.2. As earlier, the PC possesses 42 layers of the same dimensionless length *d*_1_ = *d*_2_ = 0.395. The alternating PC layers possess close dielectric permittivity *ε*_1_ = 1.7, *ε*_2_ = 1.5 and focusing nonlinearity *α*_1_ = −10^−3^, *α*_2_ = −1.5 ⋅ 10^−3^. The dielectric permittivity values are measured in the units of the ambient medium permittivity, so that *ε*_3_ = 1. The ambient medium is a linear one (*α*_0_ = 0) or possesses a focusing nonlinearity which is two times smaller than the odd PC layers nonlinearity: *α*_0_ = −5 ⋅ 10^−4^. The ratio of the laser radiation frequency and the periodic structure frequency is Ω = 7.5858. The corresponding physical parameters are: the light wavelength *λ* = 1 *μm*; the thickness of the layers *d*_1_ = *d*_2_ = 3*μm*, and characteristic wavelength of the layered structure *λ*_0_ ≈ 7.5858 *μm*. Power density of optical radiation is equal to a few tens MW/cm^2^. The soliton initial profile |*A*_*s*_(*z*)|^2^ is shown by the black line in the insert in Fig 6D and the initial phase distribution is shown in the same figure by the red line. At the time moment t = 0, the soliton is formed inside the PC and is spreading over about 20 layers.

Let us stress that relatively low laser pulse intensities and close values of dielectric permittivity for the alternating layers allows experimental observation of the soliton modes under discussion. Keeping this in mind, below we consider the soliton propagation with transverse wave number Ω_*z*_ = 0.0663. At this wave number, the moving soliton is formed in the PC with a linear ambient medium (Fig 6B, red line), while if a laser pulse propagates in the PC with focusing ambient medium, then an oscillating surface soliton is formed (Fig 6B, black line). It occupies from 14 to 7 PC layers depending on its oscillating position at the right boundary, and it penetrates into the ambient medium at the similar distance (Fig 6D and 6E).

The process of oscillating soliton formation at the right PC boundary is similar to the previously considered cases. After each reflection, the soliton velocity damping and intensity decreasing occur (Fig 6A and 6B). In the case of the focusing medium, the soliton velocity damping results in the soliton localization at the right boundary after two reflections from the left boundary. Note that the velocity of the soliton motion between the reflections strongly depends on the ambient medium properties: it is approximately one and a half times larger for the linear ambient medium than for the medium with focusing nonlinearity. Moreover, the part of the energy that temporarily or completely leaves the PC as the soliton reflects from the PC boundary, also depends on the type and the strength of the ambient medium properties: it is smaller for the linear medium (Fig 6C).

## 4. Aberrated soliton formation near the boundary of a 2-D nonlinear layered photonic crystal

The parameters and variables of Eq (2) for the laser pulse interaction with 2-D PC, consisting of spheres (Fig 1B) are the following:

$$\begin{array}{l} a_{z}=c\tau ,\;D_{x}=-D_{z}\frac{\lambda_{0}^{2}}{\lambda_{0x}^{2}},\;\lambda_{0}=(2R+d_{z})\sqrt{\varepsilon_{1}}+(2R+d_{x})\sqrt{\varepsilon_{2}},\;\lambda_{x0}=(2R+d_{x})\sqrt{\varepsilon_{1}}+(2R+d_{x})\sqrt{\varepsilon_{2}}\\ R\to \frac{R}{\lambda_{0}},\;a_{z}\to \frac{a_{z}}{\lambda_{0}},\;a_{x}\to \frac{a_{x}}{\lambda_{x0}},\;z\to \frac{z}{\lambda_{0}},\;x\to \frac{x}{\lambda_{x0}},\;t\to t\frac{c}{\lambda_{0}},\;L_{z}\to \frac{L_{z}}{\lambda_{0}},\;L_{x}\to \frac{L_{x}}{\lambda_{x0}},\;L_{t}\to \frac{cL_{t}}{\lambda_{0}}. \end{array}$$ (7)

Above parameter *a*_*x*_ and *a*_*z*_ characterize the laser beam profile on the x- and z-coordinate, respectively. The PC is characterized by the sphere radius *R*_*s*_, the distance between the spheres in the x-coordinate *d*_*x*_ and the distance between the spheres in the z-coordinate *d*_*z*_. The other dimensionless parameters are specified in Eq (3) Functions *ε*(*z*,*x*), *α*(*z*,*x*) are the following:

$$(\varepsilon (z,x),\alpha (z,x))=\left\{ \begin{array}{l} (\varepsilon_{0},\alpha_{0}),\;(z,x)\;\mathrm{is}\;\mathrm{before}\;\mathrm{the}\;\mathrm{PC}\\ (\varepsilon_{1},\alpha_{1}),\;(z,x)\;\mathrm{is}\;\mathrm{inside}\;\mathrm{the}\;\mathrm{sphere},\;\mathrm{odd}\;\mathrm{photonic}\;\mathrm{layers}\\ (\varepsilon_{2},\alpha_{1}),\;(z,x)\;\mathrm{inside}\;\mathrm{the}\;\mathrm{sphere},\;\mathrm{even}\;\mathrm{photonic}\;\mathrm{layers}\\ (\varepsilon_{3},\alpha_{2}),\;(z,x)\;\mathrm{is}\;\mathrm{in}\;\mathrm{the}\;\mathrm{substrate}\;\mathrm{between}\;\mathrm{the}\;\mathrm{spheres}\\ (\varepsilon_{4},\alpha_{0}),\;(z,x)\;\mathrm{is}\;\mathrm{in}\;\mathrm{the}\;\mathrm{lateral}\;\mathrm{substrate} \end{array}\right.$$

Negative value of *α*_0_, *α*_1_ and *α*_2_ results in the laser beam self-focusing. *ε*_1_, *ε*_2_, *ε*_3_ and *ε*_4_ are the dielectric permittivity of the spheres in the odd photonic layers, in the even photonic layers, in the substrate beside the PC and in the lateral substrate, respectively; *ε*_0_ = 1 is a dielectric permittivity of the substrate before the PC.

Initial conditions is written in the form

$$A(z,x,t)|{}_{t=0}=A_{0}\exp(-{\left(x-L_{xc}\right)}^{2}/a_{x}{}^{2})\exp(-{\left(z-L_{zc}\right)}^{2}/a_{z}{}^{2}-i2\pi \Omega (z-L_{zc})),$$

where *L*_*xc*_ and *L*_*zc*_ are the center at the initial time moment.

**Fig 7:**
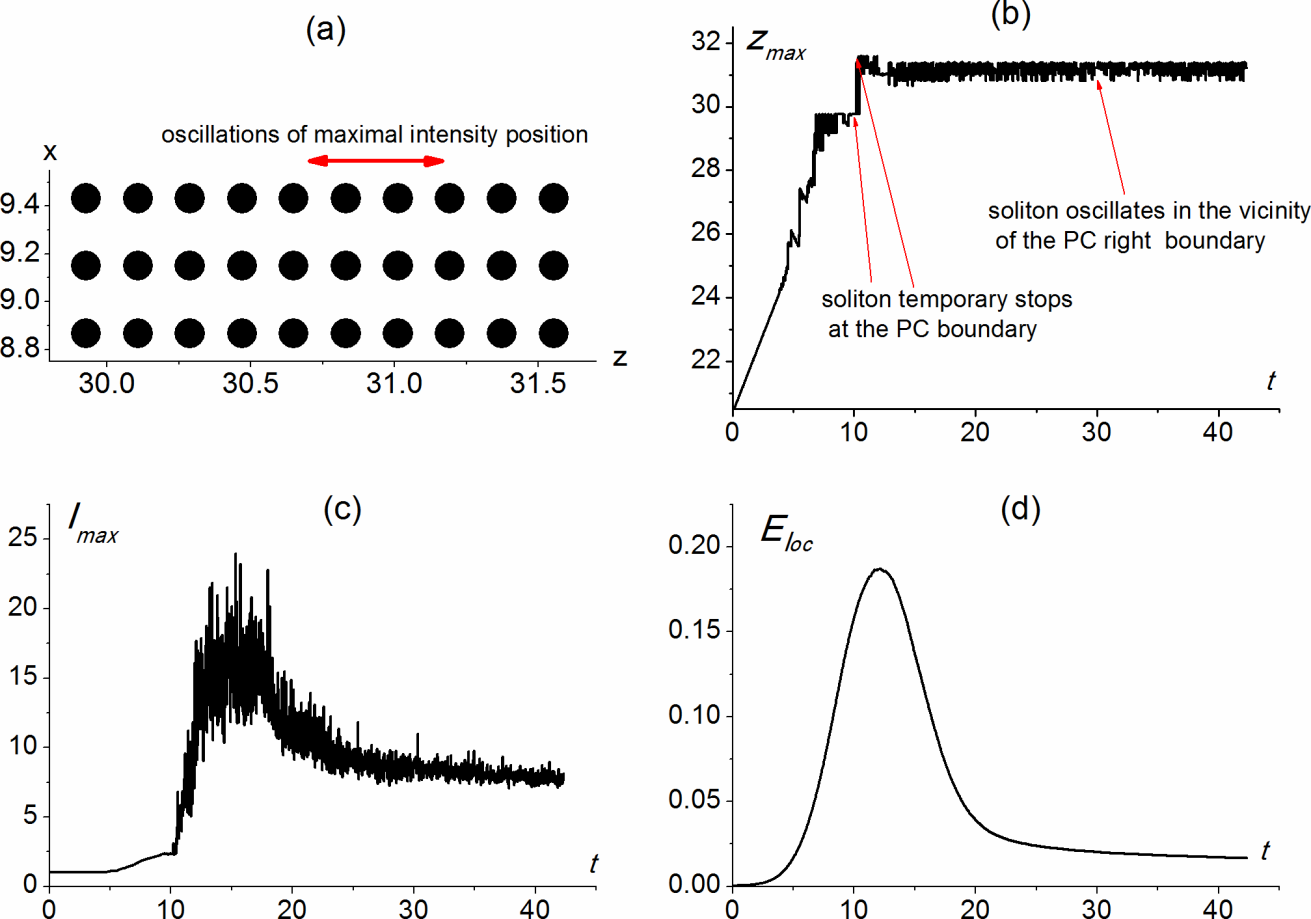
Scheme of 2-D PC (a); maximal intensity position on the z-coordinate (b), maximal intensity (c) and localized energy (d) versus time calculated for parameters equal to *α*_0_ = 0, *α*_1_ = 0, *α*_2_ = -0.6822, *a*_*x*_ = 1.826.

**Fig 8:**
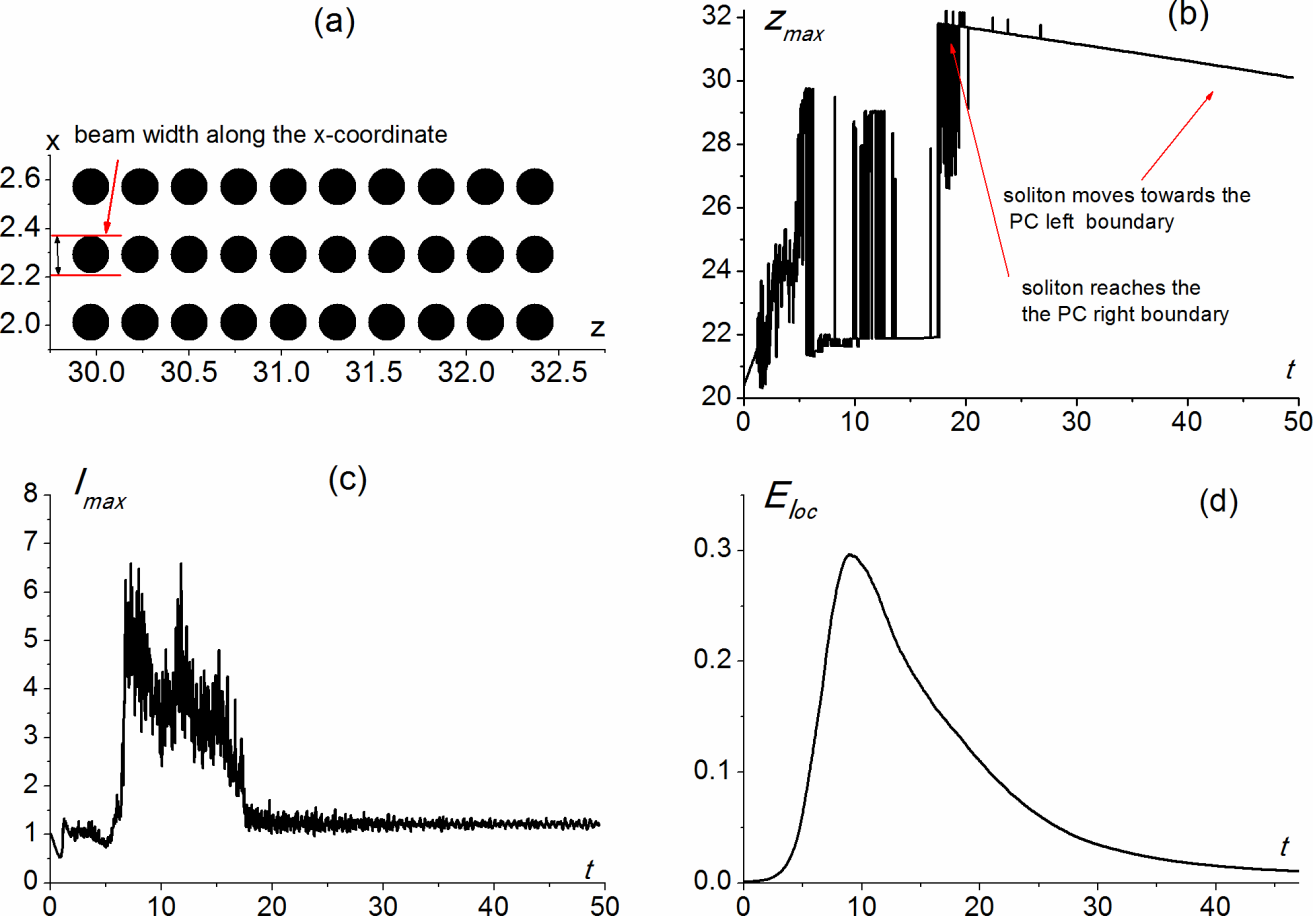
Scheme of 2-D PC (a); position of maximal intensity (b), value of maximal intensity (c) and localized energy (d) versus time. Dimensionless parameters values are *α*_0_ = 1.421, *α*_1_ = 0, *α*_2_ = -6.822, *a*_*x*_ = 0.09312.

**Fig 9:**
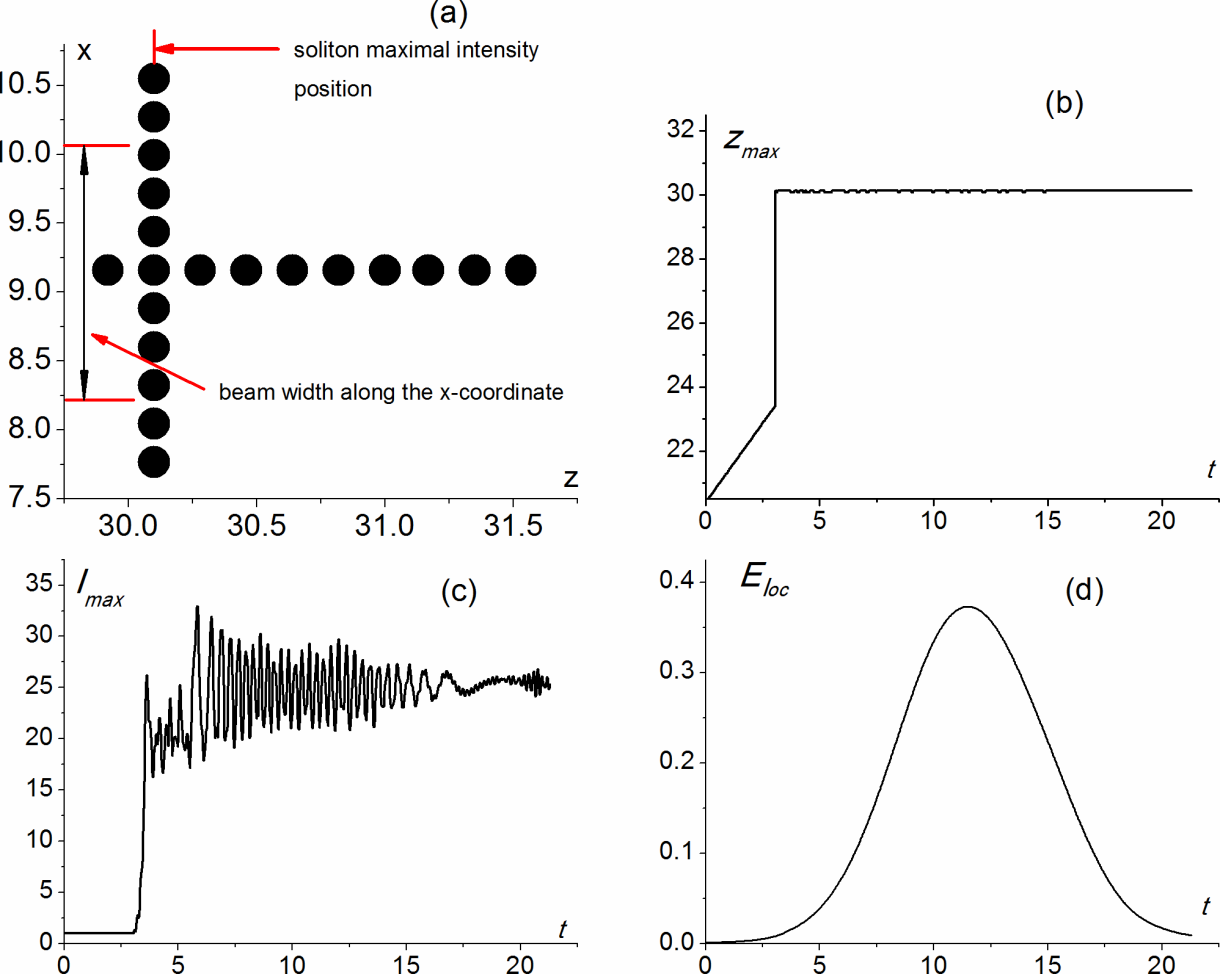
Scheme of 2-D PC (a); maximal intensity position (b), maximal intensity (c) and localized energy (d) versus time calculated for parameters equal to *α*_0_ = 0.01421, *α*_1_ = *α*_2_ = -0.06822, *a*_*x*_ = 1.826.

In our computer simulations we consider two types of PC–rectangular and cruciform—with the distances between the spheres along the z-coordinate and the x-coordinate equal to the sphere radius: *R*_*s*_ = *d*_*x*_ = *d*_*z*_ = 0.5 *μm* (Figs 7A–9A). The dielectric permittivity inside and outside the spheres are the following: *ε*_1_ = 2, *ε*_2_ = 5, *ε*_3_ = 3, *ε*_4_ = 8. The wavelength of the incident laser radiation is equal to *λ* = 0.5 *μm* and the pulse duration is equal to *τ* = 0.1 ps, so the beam width along the z-coordinate is *a*_*z*_ = *cτ* = 30 *μm*. Characteristic wavelengths of the layered structure according to (7) are *λ*_0_ = *λ*_*x*0_ ≈ 5.475 *μm*. Corresponding dimensionless parameters are: *R*_*s*_ = *d*_*x*_ = *d*_*z*_ = 9.312 ⋅ 10^−2^, *a*_*z*_ = 5.479 and Ω = 10.95.

In all cases, the incident beam is located before the PC and propagates along the z-coordinate towards the PC. The PC length along the z-coordinate is 15.50 *μm*, the PC width along the x-coordinate is 5 *μm* for the rectangular PC and 16.97 *μm* for the cruciform PC. The incident beam center along the z-coordinate coincides with the PC center.

Figs 7 and 8 show the light energy localization for the rectangular PC consisting of ten sphere rows along the z-coordinate and three sphere rows along the x-coordinate.

Light energy localization occurs near the right boundary of the rectangular PC (Fig 7) if the spheres posses the focusing nonlinearity (*α*_1_ = -0.6822), while the medium between the spheres and outside the spheres are linear one: *α*_0_ = *α*_2_ = 0, and the beam radius along the x-coordinate is two times larger than the PC size along the x-coordinate (*a*_*x*_ = 1.826, *L*_*xpc*_ = 0.91324). These dimensionless parameters correspond to the beam radius along the x-coordinate equal to 10 *μm* and input intensity of optical radiation equal to several GW/cm^2^.

We believe that light localization occurs due to the soliton formation near the right PC face. Indeed, at about time moment t = 6, the beam reaches the PC face and, for about next 4 dimensionless time units, it is located at the left boundary of the PC (z = 29.8), but then it rushed to the right boundary (z = 31.6). For about 3 dimensionless time units the soliton maximum intensity position oscillates between the first and the fourth layer from the right, and then the range of its oscillations moves to the 2^nd^ - 5^th^ layers from the right (the range of oscillations is shown by an arrow in Fig 7A). Maximum intensity of the soliton is 7.5 times greater than the intensity of the incident pulse (Fig 7C). The energy, localized in the PC, is about 1.6%, while it is 10 time larger at *t* ≈ 12 when the soliton starts oscillating between the first and the fourth layer from the right (Fig 7D).

Light energy localization also takes place if the medium inside the PC spheres is linear (*α*_1_ = 0), while the medium between the spheres posses the strong focusing nonlinearity (*α*_2_ = -6.822) and the medium outside the PC is a defocusing one (*α*_0_ = 1.421) (Fig 8). In this case, the input intensity of the laser radiation is several tens GW/cm^2^, which is ten times larger than in the previous case (shown in Fig 7), and the laser beam radius along the x-coordinate is equal to the sphere radius: 0.5 *μm* (*a*_*x*_ = 0.09132).

After the beam falls on the PC face, it passes through the PC from the left to the right boundary. The soliton reaches the right boundary (*t* ≈ 20), so that the maximal intensity is achieved at the third row of sphere from the right (*z* ≈ 31.8). Then the soliton reflects from the PC right boundary and then slowly moves towards the PC left boundary. At the end of the time interval under consideration, the soliton is still inside the PC (the position of its maximal intensity is about *z* ≈ 30.11). While localizing inside the PC, the soliton maximal intensity is about one dimensionless unit and equals to the incident beam maximal intensity (Fig 8C). The amount of optical energy, localized in the PC at the time *t* ≈ 45, is about one 1% (Fig 8D), while at the time *t* ≈ 20, when the soliton reaches the right PC boundary, it is about 10%. Nevertheless, the pulse intensity inside the PC is about unity during the time interval 20<*t*<45.

For the cruciform PC (Fig 9A), the light energy is localized at the transverse row if the PC spheres and the medium between the spheres possess the same focusing nonlinearity *α*_1_ = *α*_2_ = -0.06822, the ambient medium is a defocusing one *α*_0_ = 0.01421 and the incident beam radius is about two times smaller than the PC size in the x-coordinate (*a*_*x*_ = 1.826, *L*_*xpc*_ = 3.105). The input intensity of laser radiation is about 0.1 GW/cm^2^, the PC length and beam radius along the x-coordinate are 16.97 *μm* and 10 *μm*, respectively. In this case, the incident beam falls on the PC face and stops at the second row consisting of 11 spheres located along the x-coordinate (Fig 9B). The localized beam intensity in about 25 times higher than the intensity of the incident beam. The localized energy is the highest at *t* ≈ 12. At that time the soliton is already stopped. Then, the localized energy gradually decreases, reaching the value 1% at the end of the time interval under consideration (Fig 9C).

To summarize, in this Section we have shown the possibility for laser radiation localization in a 2-D PC consisting of spheres for various PC configurations and laser radiation parameters. In particular, we considered three different cases of the relationship between the PC width and the laser beam radius and two different types of PC–the cruciform PC and the rectangular PC. In all the considered cases, it is shown that laser energy localization occurs.

## 5. Conclusions

We have investigated the influence of PC and laser radiation properties on the formation of the novel type surface soliton (aberrated soliton) in 1-D PC and the possibility of this type soliton formation in 2-D PC.

In the 1-D PC case, aberrated solitons are formed from the initial soliton placed inside the PC under the perturbation of transverse component of its wave vector. The initial soliton belongs to the central area of the nonlinear PC, spreading over a number of PC layers. The surface soliton are characterized by their transverse dimensions; they cover several PC layers. The distance of the surface soliton penetrating into PC is about the distance of penetration into the substrate and reaches the length of several layers.

We have also shown the possibility for soliton formation in the 2-D PC. In this case, the incident beam falls on the PC face and stops at a PC boundary or reflects from it and changes the direction of motion.

It should be stressed that the possibility for the aberrated surface soliton formation was shown for low laser radiation intensity and close dielectric permittivity of the PC alternation layers, which allows us to observe these solitons in physical experiment. A particular example is a PC with focusing nonlinearity which is slightly different for alternating layers (the difference about one and a half times) and close dielectric permittivity, for example, *ε*_1_ = 1.7 and *ε*_2_ = 1.5 in the units of the ambient medium permittivity. The light wavelength can be about 1 *μm*; the thickness of the PC layers—about 3 *μm*. Power density of optical radiation can be a few tens MW/cm^2^ with pulse duration of about 80 fs -100 fs. By changing the nonlinearity of the ambient medium and the strength of the transverse perturbations of the incident beam wave vector, it is possible to control the surface soliton generation.
